# Supplementary material for: Occurrence and Distribution of Per- and Polyfluoroalkyl Substances from Multi-Industry Sources to Water, Sediments and Plants along Nairobi River Basin, Kenya
Source: Int J Environ Res Public Health. 2022 Jul 23;19(15):8980. doi: 10.3390/ijerph19158980 (PMC9331875; doi:10.3390/ijerph19158980)
Supplement: Supplementary file 1 [file ijerph-19-08980-s001.zip › ijerph-1804595-supplementary.pdf]

Occurrence and Distribution of Per- and Polyfluoroalkyl Substances from Multi-Industry Sources to Water, Sediments and Plants along Nairobi River Basin, Kenya

Chirikona Nyangweso Flora[1*], Natalia Soares Quinete[2], Jesleen Gonzalez[2], Mutua Gershom [1], Kimosop Selly [1], Orata Francis[1]

[1]Department of Pure and Applied Chemistry, Masinde Muliro University of Science and Technology. P.O Box 190 Kakamega 50100

[2]Department of Chemistry & Biochemistry Institute of Environment, Freshwater Resources Division Florida International University, 3000 NE 151st street, Biscayne Bay Campus North Miami, FL33181, USA

*Corresponding author E-mail: chirikonaflory@gmail.com.Tel: +254-723789441

Supplementary information

Table S1: List of 30 PFAS with their internal standards applied for quantitation and list of 24 PFAS in the secondary standard.

Abbreviation	Compound Name	Molecular Formula	Molecular Weight	I.S.	Secondary Standard
4-2 FTS	Sodium 1H,1H,2H,2H-perfluoro-1-hexanesulfonate	$C_6H_5F_9O_3S$	328.15	M2 4-2 FTS	✓
6-2FTS	Sodium 1H,1H,2H,2H-perfluoro-1-octanesulfonate	$C_8H_5F_{13}O_3S$	428.17	M2 6-2FTS	✓
8-2 FTS	Sodium 1H,1H,2H,2H-perfluoro-1-decanesulfonate	$C_{10}H_5F_{17}O_3S$	528.18	M2 8-2 FTS	✓
ADONA	Sodium dodecafluoro-3H-4,8-dioxanonanoate	$C_{10}H_{11}N_4NaO_5S$	322.27	M2 6-2FTS	
FBSA	Perfluoro-1-butanesulfonamide	$C_4H_2F_9NO_2S$	299.11	M GenX	
FHxSA	Perfluoro-1-hexanesulfonamide	$C_6H_2F_{13}NO_2S$	399.13	M8 PFOA	
FOSA	Perfluoro-1-octanesulfonamide	$C_8H_2F_{17}NO_2S$	499.14	M8 FOSA	
HFPO-DA	2,3,3,3-Tetrafluoro-2-(1,1,2,2,3,3,3-heptafluoropropoxy)-propanoic acid	$C_6HF_{11}O_3$	330.05	M GenX	
N-EtFOSAA	N-ethylperfluoro-1-octanesulfonamidoacetic acid	$C_{12}H_8F_{17}NO_4S$	585.23	d5 N-EtFOSAA	✓
N-MeFOSAA	N-methylperfluoro-1-octanesulfonamidoacetic acid	$C_{11}H_6F_{17}NO_4S$	571.21	d3 N-MeFOSAA	✓
PFBA	Perfluoro-n-butanoic acid	$C_4HF_7O_2$	214.04	MPFBA	✓
PFBS	Potassium perfluoro-1-butanesulfonate	$C_4HF_9O_3S$	300.10	M3 PFBS	✓
PFDA	Perfluoro-n-decanoic acid	$C_{10}HF_{19}O_2$	514.08	M6 PFDA	✓
PFDoA	Perfluoro-n-dodecanoic acid	$C_{12}HF_{23}O_2$	614.10	M PFDoA	✓
PFDS	Sodium perfluoro-1-decanesulfonate	$C_{10}HF_{21}O_3S$	600.14	M7 PFUdA	✓
PFHpA	Perfluoro-n-heptanoic acid	$C_7HF_{13}O_2$	364.06	M4 PFHpA	✓
PFHpS	Sodium perfluoro-1-heptanesulfonate	$C_7HF_{15}O_3S$	450.12	M3 PFHxS	✓
PFHxA	Perfluoro-n-hexanoic acid	$C_6HF_{11}O_2$	314.05	M5 PFHxA	✓
PFHxS	Potassium perfluorohexanesulfonate	$C_6HF_{13}O_3S$	400.11	M3 PFHxS	✓

PFNA	Perfluoro-n-nonanoic acid	$C_9HF_{17}O_2$	464.08	M3 PFHxS	✓
PFNS	Sodium perfluoro-1-nonanesulfonate	$C_9HF_{19}O_3S$	550.14	M8 PFOS	✓
PFOA	Perfluoro-n-octanoic acid	$C_8HF_{15}O_2$	414.07	M8 PFOA	✓
PFONS	Potassium 9-chlorohexadecafluoro-3-oxanonane-1-sulfonate	$C_8ClF_{16}KO_4S$	570.67	M8 PFOS	
PFOS	Potassium perfluorooctanesulfonate	$C_8HF_{17}O_3S$	500.13	M8 PFOS	✓
PFOUdS	Potassium 11-chloroeicosafluoro-3-oxaundecane-1-sulfonate	$C_{10}ClF_{20}KO_4S$	670.69	M PFDoA	
PFPeA	Perfluoro-n-pentanoic acid	$C_5HF_9O_2$	264.05	M5 PFPeA	✓
PFPeS	Sodium perfluoro-1-pentanesulfonate Potassium	$C_5HF_{11}O_3S$	350.11	M GenX	✓
PFTeDA	Perfluoro-n-tetradecanoic acid	$C_{14}HF_{27}O_2$	714.11	M2 PFTeDA	✓
PFTrDA	Perfluoro-n-tridecanoic acid	$C_{13}HF_{25}O_2$	664.11	M2 PFTeDA	✓
PFUdA	Perfluoro-n-undecanoic acid	$C_{11}HF_{21}O_2$	564.09	M7 PFUdA	✓

Table S2: The LC gradient conditions

Time (Min)	A [%]	B %	Flow mL/min
0.00	90	10	0.4
8.00	5	95	0.4
11.00	5	95	0.4
12.00	90	10	0.4

Table S3 Ms parameters for LC- MS/MS Analysis

Parameters	Settings
Ms Aquisition	Dynamic MRM
Cycle time	500 ms
Ion source	ESI negative
Drying gas Temperature and Flow	150 0c and 10L/Min
Sheath gas temperature and flow	300 and 10L/Min
Capillary	2000
Nozzle Voltage	0 V

Table S4. Summary of the MRM method for the analysis of PFAS

Compound Name	ISTD	Precursor Ion	Product Ions	Retention Time (min)	Delta Retention Time	Fragmentor	Collision Energy	Cell Accelerator Voltage
4-2 FTS		327	80.9	5.69	3	125	32	5
6-2FTS		427	406.8; 79.9	6.95	3	125	24	5
8-2 FTS		527	506.8; 80.9	7.65	3	170	28	5
Adona		377	250.9	6.54	3	70	10	5
d3 N-MeFOSAA	✓	573	419	8.1	3	115	20	5
d5 N-EtFOSAA	✓	589	419	8.18	3	115	20	5
FBSA		298	77.9	6.83	3	100	28	5
FHxSA		398	77.9	7.95	3	100	28	5
FOSA		497.9	77.9	8.63	3	125	36	5
GenX		285	169	5.88	3	108	12	5
M GenX	✓	287	185	5.88	3	108	20	5
M PFDoA	✓	615	570	8.21	3	79	8	5
M2 4-2 FTS	✓	329	81	5.66	3	125	28	5
M2 6-2FTS	✓	429	81	6.94	3	125	32	5
M2 8-2 FTS	✓	529	81	7.66	3	170	36	5
M2 PFTeDA	✓	715	670	8.72	5	100	13	5
M3 PFBS	✓	302	99	4.99	3	100	33	5
M3 PFHxS	✓	402	99	6.57	3	100	41	5
M4 PFHpA	✓	367	322	6.52	3	72	8	5
M5 PFHxA	✓	318	273	5.79	3	70	8	5
M5 PFPeA	✓	268	223	4.35	3	60	4	5
M6 PFDA	✓	519	474	7.7	3	81	8	5
M7 PFUdA	✓	570	525	7.98	3	73	8	5
M8 FOSA	✓	506	78	8.63	3	125	36	5

M8 PFOA	✓	421	376	7.01	3	69	8	5
M8 PFOS	✓	507	99	7.37	3	100	46	5
M9 PFNA	✓	472	427	7.39	3	66	8	5
MPFBA	✓	217	172	2.18	3	60	8	5
N-EtFOSAA		584	525.9; 418.9	8.18	3	115	20	5
N-MeFOSAA		570	482.9;418.9	8.09	3	115	16	5
PFBA		213	168.9	2.18	3	60	8	5
PFBS		298.9	98.9;80	4.98	3	100	33	5
PFDA		513	469	7.7	3	81	8	5
PFDoA		613	569; 268.7	8.21	3	79	8	5
PFDS		598.9	99	7.93	3	100	56	5
PFDS		598.9	80	7.93	3	100	88	5
PFHpA		362.9	319; 169	6.52	3	72	5	5
PFHpS		448.9	98.7; 79.7	7.01	3	100	44	5
PFHxA		313	268.9	5.79	3	70	4	5
PFHxS		398.9	99;80	6.56	3	100	41	5
PFNA		463	419;169	7.39	3	66	8	5
PFNS		548.9	98.9; 79.9	7.67	3	165	48	5
PFOA		413	369;169	7.01	3	69	8	5
PFONS		530.9	350.9	7.44	3	100	17	5
PFOS		498.9	99; 80	7.37	3	100	46	5
PFOUDS		630.9	450.9	7.97	3	100	17	5
PFPeA		263	218.9	4.3	3	60	4	5
PFPeS		348.9	98.9;79.9	5.97	3	135	36	5
PFTeDA		713	669;169	8.6	3	100	13	5
PFTrDA		663	619;169	8.41	3	91	9	5
PFUdA		563	519	7.97	3	73	8	5
4-2 FTS		327	306.9	5.69	3	125	20	5

Table S5. Method Validation results for PFAS.

**Compounds**	**MDL (ng/L) water**	**MDL (ng/g) sediments and plants**
PFBA	0.001	0.1
FBSA	0.01	0.1
PFBS	0.01	0.2
PFPeA	0.05	2
PFPeS	0.003	0.4
PFHxA	0.01	0.4
FHxSA	0.02	0.4
PFHxS	0.01	0.1
4-2 FTS	0.03	0.4
Adona	0.01	0.4
GenX	0.03	0.1
PFHpA	0.08	0.2
PFHpS	0.04	0.4
PFOA	0.01	0.2
FOSA	0.03	0.2
PFOS	0.01	0.2
6-2 FTS	0.22	0.1
PFONS	0.01	0.4
PFNA	0.02	0.1
N-MeFOSAA	0.49	0.2
N-EtFOSAA	0.27	
PFNS	0.01	0.2
PFDA	0.02	0.1
PFDS	0.09	
8-2 FTS	0.002	1
PFUdA	0.21	1
PFDoA	0.04	
PFTrDA	0.54	0.4

Table S6. PFAS abbreviation list. Long chain PFAS are characterized by a carbon chain greater than or equal to 8 whereas with short-chain PFAS are defined as the ones with a carbon chain less than 8.

Compound Class	Compound Name	Abbreviation	Structure	Chain Length
**Perfluoroalkyl carboxylic acid (PFCA)**	Perfluorobutanoic acid	PFBA	C_3F_7COOH	Short
	Perfluoropentanoic acid	PFPeA	C_4F_9COOH	Short
	Perfluorohexanoic acid	PFHxA	$C_5F_{11}COOH$	Short
	Perfluoroheptanoic acid	PFHpA	$C_6F_{13}COOH$	Short
	Perfluorooctanoic acid	PFOA	$C_7F_{15}COOH$	Long
	Perfluorononanoic acid	PFNA	$C_8F_{17}COOH$	Long
	Perfluorodecanoic acid	PFDA	$C_9F_{19}COOH$	Long

	Perfluoroundecanoic acid	PFUdA	$C_{10}F_{21}COOH$	Long
	Perfluorododecanoic acid	PFDoA	$C_{11}F_{23}COOH$	Long
	Perfluorotridecanoic acid	PFTrDA	$C_{12}F_{25}COOH$	Long
	Perfluorotetradecanoic acid	PFTeDA	$C_{13}F_{27}COOH$	Long
**Perfluoroalkyl sulfonic acid (PFSA)**	Perfluorobutane sulfonic acid	PFBS	$C_4F_9SO_3H$	Short
	Perfluoropentane sulfonic acid	PFPeS	$C_5F_{11}SO_3H$	Short
	Perfluorohexane sulfonic acid	PFHxS	$C_6F_{13}SO_3H$	Short
	Perfluoroheptane sulfonic acid	PFHpS	$C_7F_{15}SO_3H$	Short
	Perfluorooctane sulfonic acid	PFOS	$C_8F_{17}SO_3H$	Long

	Perfluorononane sulfonic acid	PFNS	$C_9F_{19}SO_3H$	Long
	Perfluorodecane sulfonic acid	PFDS	$C_{10}F_{21}SO_3H$	Long
**Fluorotelomer sulfonic acid (FTS)**	4:2 Fluorotelomer sulfonic acid	4-2 FTS	$C_4F_9C_2H_4SO_3H$	Short
	6:2 Fluorotelomer sulfonic acid	6-2 FTS	$C_6F_{13}C_2H_4SO_3H$	Long
	8:2 Fluorotelomer sulfonic acid	8-2 FTS	$C_8F_{17}C_2H_4SO_3H$	Long
**Perfluoroether carboxylic acid (PFECA)**	Perfluoro-2-methyl-3-oxahexanoic acid	GenX	$C_6F_{11}O_3H$	Short
	4,8-Dioxa-3H-perfluorononanoic acid	Adona	$C_7H_2F_{12}O_4$	Short

**Perfluoroalkane sulfonamido acetic acid (FOSAA)**	N-Methyl perfluorooctane sulfonamido acetic acid	N-MeFOSAA	$C_8F_{17}SO_2N[CH_3]CH_2COOH$	Long
	N-Ethyl perfluorooctane sulfonamido acetic acid	N-EtFOSAA	$C_8F_{17}SO_2N[C_2H_5]CH_2COOH$	Long
**Perfluoroalkane sulfonamide (PFOSA)**	Perfluorobutanesulfonamide	FBSA	$C_4F_9SO_2NH_2$	Short
	Perfluorohexanesulfonamide	FHxSA	$C_6F_{13}SO_2NH_2$	Short
	Perfluorooctanesulfonamide	FOSA	$C_8F_{17}SO_2NH_2$	Long

Table S7: Range, mean, total concentration and the detection frequency of PFAS in water in the eight sampling points along the Nairobi River.

PFAS	Range	Mean	Total ng/L	Detection frequency
PFBA	0.74-4.79	1.69	13.52	100%
PFPeA	0.28-3.92	1.13	9.07	100%
PFBS	0.01- 1.0	0.15	1.23	100%
4-2 FTS	0.01- 0.05	0.02	0.18	100%
PFHxA	0.15- 3.0	0.74	5.9	100%
PFPeS	0.05- 0.11	0.06	0.12	25%
GenX	0.01- 0. 12	0.06	0.53	100%
PFHpA	0.13- 2.41	0.60	4.80	100%
Adona	<LOD- 0.01	0.01	0.02	50%
PFHxS	<LOD- 0.47	0.11	0.11	62.5%
FBSA	0.01- 0.06	0.03	0.23	100%
6 – 2 FTS	0.77- 5.53	3.66	29.31	100%
PFOA	0.16- 3.01	0.74	5.90	100%
PFHpS	<LOD- 0.01	0.01	0.01	75%
PFOS	0.01-1.37	0.23	1.83	100%
PFNA	0.06- 1.39	0.34	2.69	100%
PFONs	<LOD- 0.02	0.01	0.06	75%
PFNs	<LOD- 0.09	0.01	0.01	12.5%
8-2 FTs	0.02- 30.11	0.03	0.24	100%
PFDA	0.04- 0.67	0.19	1.50	100%
PFDS	0.01- 1.30	0.22	1.78	100%
PFOUDS	<LOD- 0.38	0.15	0.50	37.5%
PFUdA	0.05- 13. 22	4.9	39.19	100%
FH_xSA	0.002-0.013	0.01	0.06	87.5%
N-MeFOSAA	<LOD-0.22	0.06	0.39	87.5%

N-EtFOSAA	<LOD-0.24	0.06	0.31	37.5%
PFDoA	0.03- 2.40	0.76	6.06	100%
PFTrDA	<LOD-0.02	0.001	0.02	87.5%
PFTeDA	<LOD-0.54	0.31	2.17	75%
FOSA	0.01-0.1	0.04	0.023	100%

Table S8: Range, mean, total concentration and the detection frequency of PFAS in sediments

PFAS	Range	Mean	Total ng/g	Detection frequency
PFBA	0.55 - 5.47	2.85	22.81	100%
PFPeA	1.58 - 10.91	4.75	37.98	100%
PFBS	0.12 - 6.18	2.0	16.01	100%
4-2 FTS	< MDL	0.00	0.00	0%
PFHxA	0.46 - 1.50	0.81	6.51	100%
PFHxS	0 - 3.70	1.49	10.42	85.5%
PFHpA	0.17- 0.84	0.42	3.40	100%
ADONA	0 - 0.77	0.25	1.49	75%
6-2 FTS	1.62	1.62	1.62	12.5%
PFHpS	1.69	1.69	1.69	12.5%
PFOA	0.88 - 13.60	4.95	39.62	100%
FBSA	0- 0.21	0.17	1.19	85.5%
PFOS	2.44- 11.74	5.15	41.16	100%
PFONS	0 - 0.2741	0.20	0.80	50%
PFNA	0.053 -1.05	0.40	3.16	100%
PFNS	0 -7.17	5.17	31.03	75%
8-2 FTS	0.03- 0.87	0.36	2.88	100%
PFDA	0.47- 3.76	1.43	11.43	100%
PFDS	1.55	1.56	1.56	12.5%
PFOUDS	0.20- 0.36	0.26	0.52	25%
PFUdA	0.33- 1.65	1.00	8.01	100%
FHxSA	0- 0.02	0.09	0.68	100%
PFDoA	0.30- 1.35	0.79	6.31	100%
N.MeFOSAA	0.52 -1.66	1.00	8.03	100%
N-EtFOSAA	0.44- 1.51	0.87	6.94	100%
PFTrDA	0.21- 1.01	0.41	3.31	100%
PFTeDA	0.10- 0.75	0.40	3.18	100%
FOSA	0- 0.60	0.39	3.17	100%

Table S9: Range, mean, total concentration and the detection frequency of PFAS in plants

PFAS	Range	Mean	Total	Detection frequency
PFBA	<MDL- 15	4.71	28.23	83.33%
PFPeA	2.75 -9.00	5.41	32.47	100%
PFBS	<MDL -2.3	0.11	0.71	83.33%
4-2 FTS	<MDL- 0.8	0.13	0.80	16.67%
PFHxA	<MDL- 2.8	0.87	5.21	83.33%
PFHxS	<MDL- 2.04	0.85	3.79	33.33%
PFHpA	0.20-1.14	0.61	3.67	100%
ADONA	<MDL- 0.12	0.11	0.14	33.33%
6-2 FTS	<MDL-2.90	0.48	2.90	16.67%
PFHpS	<MDL	0	0	0%
PFOA	2.85- 5.60	4.89	29.33	100%
FBSA	<MDL – 0.19	0.03	0.19	16.67%
PFOS	0.02- 2.66	0.95	5.68	100%
PFONS	<MDL – 0.21	0.09	0.56	50%
PFNA	<MDL – 1.04	0.26	1.53	33.33%
PFNS	<MDL- 0.53	0.09	0.54	16.67%
8-2FTS	<MDL -0.86	0.24	1.45	16.67%
PFDA	<MDL -1.29	0.41	2.48	83.33%
PFDS	<MDL- 0.91	0.15	0.91	16.67%
PFOUDS	<MDL	0	0	0
PFUdA	<MDL -1.32	0.38	2.25	66.67%
FHxSA	<MDL – 0.12	0.02	0.12	16.67%
PFDoA	0.05 – 0.37	0.20	1.22	100%
N.MeFOSAA	<MDL-0.20	0.28	1.67	66.67%
N-EtFOSAA	<MDL -0.003	0.0004	0.003	16.67%
PFTrDA	<MDL- 1.92	0.42	2.53	66.67%
PFTeDA	<MDL- 0.20	0.12	0.71	83.33%
FOSA	<MDL- 1.02	0.17	1.02	16.67%
